# Fucoxanthin, a Marine Carotenoid, Attenuates *β*-Amyloid Oligomer-Induced Neurotoxicity Possibly via Regulating the PI3K/Akt and the ERK Pathways in SH-SY5Y Cells

**DOI:** 10.1155/2017/6792543

**Published:** 2017-08-08

**Authors:** Jiajia Lin, Jie Yu, Jiaying Zhao, Ke Zhang, Jiachen Zheng, Jialing Wang, Chunhui Huang, Jingrong Zhang, Xiaojun Yan, William H. Gerwick, Qinwen Wang, Wei Cui, Shan He

**Affiliations:** ^1^Ningbo Key Laboratory of Behavioral Neuroscience, Zhejiang Provincial Key Laboratory of Pathophysiology, School of Medicine, Ningbo University, Ningbo 315211, China; ^2^Li Dak Sum Yip Yio Chin Kenneth Li Marine Biopharmaceutical Research Center, Ningbo University, Ningbo 315211, China; ^3^Center for Marine Biotechnology and Biomedicine, Scripps Institution of Oceanography, and Skaggs School of Pharmacy and Pharmaceutical Sciences, University of California San Diego, La Jolla, CA 92037, USA

## Abstract

Alzheimer's disease (AD), the most common neurodegenerative disorder, is characterized by neurofibrillary tangles, synaptic impairments, and loss of neurons. Oligomers of *β*-amyloid (A*β*) are widely accepted as the main neurotoxins to induce oxidative stress and neuronal loss in AD. In this study, we discovered that fucoxanthin, a marine carotenoid with antioxidative stress properties, concentration dependently prevented A*β* oligomer-induced increase of neuronal apoptosis and intracellular reactive oxygen species in SH-SY5Y cells. A*β* oligomers inhibited the prosurvival phosphoinositide 3-kinase (PI3K)/Akt cascade and activated the proapoptotic extracellular signal-regulated kinase (ERK) pathway. Moreover, inhibitors of glycogen synthase kinase 3*β* (GSK3*β*) and mitogen-activated protein kinase (MEK) synergistically prevented A*β* oligomer-induced neuronal death, suggesting that the PI3K/Akt and ERK pathways might be involved in A*β* oligomer-induced neurotoxicity. Pretreatment with fucoxanthin significantly prevented A*β* oligomer-induced alteration of the PI3K/Akt and ERK pathways. Furthermore, LY294002 and wortmannin, two PI3K inhibitors, abolished the neuroprotective effects of fucoxanthin against A*β* oligomer-induced neurotoxicity. These results suggested that fucoxanthin might prevent A*β* oligomer-induced neuronal loss and oxidative stress via the activation of the PI3K/Akt cascade as well as inhibition of the ERK pathway, indicating that further studies of fucoxanthin and related compounds might lead to a useful treatment of AD.

## 1. Introduction

Alzheimer's disease (AD), one of the most common neurodegenerative disorders affected aging populations, is characterized by the loss of functional neurons in the brain and the progressive impairments of learning and memory [1]. Although the mechanisms underlying the pathogenesis of AD are not clearly elucidated, recent studies suggested that soluble *β*-amyloid (A*β*) oligomers are the main neurotoxic species that induce neuronal death and oxidative stress at the early stage of this disease [2, 3]. A*β* oligomers, aggregated from A*β* monomers, could induce neuronal apoptosis via increasing oxidative stress, possibly as a result of altered regulation of signaling pathways [4–6]. In neurons, A*β* oligomers substantially increase the level of intracellular reactive oxygen species (ROS) [6]. Moreover, A*β* oligomers were reported to inhibit the prosurvival phosphoinositide 3-kinase (PI3K)/Akt signaling pathway, overactivate the downstream glycogen synthase kinase 3*β* (GSK3*β*), and induce neuronal death in vitro [4]. Furthermore, A*β* oligomers could act on the proapoptotic mitogen activated protein kinase (MEK)/extracellular signal-regulated kinase (ERK) pathway, leading to neuronal apoptosis [5]. Therefore, molecules which could concurrently regulate oxidative stress and signaling pathways might produce neuroprotective effects against A*β* oligomers.

Fucoxanthin, a marine carotenoid mainly extracted from edible brown seaweeds, was reported to possess beneficial biological effects, including antioxidative stress and anti-inflammation activities [7, 8]. We have previously reported that fucoxanthin can inhibit acetylcholinesterase in vitro and attenuate scopolamine-induced cognitive impairments in mice, therefore suggesting that fucoxanthin might be useful to treat AD [9]. Recent studies have shown that fucoxanthin can ameliorate A*β* monomer-induced cell death in microglia and cortical neurons [10, 11]. Moreover, fucoxanthin was reported to inhibit A*β* precursor protein-cleaving enzyme 1 (BACE-1), an enzyme that cleaves the A*β* precursor protein into A*β* monomers [12]. Taken together, these reports suggest that fucoxanthin might inhibit A*β*-mediated neurotoxicity. However, it remains unknown whether fucoxanthin can prevent A*β* oligomer-induced neurotoxicity, and moreover, how fucoxanthin is able to produce neuroprotective effects in vitro.

In this study, we have shown for the first time that fucoxanthin significantly attenuates A*β* oligomer-induced neuronal apoptosis as well as the increase of intracellular ROS in SH-SY5Y cells. We have also demonstrated that fucoxanthin concurrently produced neuroprotective effects possibly via regulating prosurvival PI3K/Akt and proapoptotic ERK pathways.

## 2. Materials and Methods

### 2.1. Chemicals and Reagents

Fucoxanthin was purified from *Sargassum horneri* according to our published procedures [9]. Briefly, a fucoxanthin-rich solution was first obtained by extraction with ethanol (ethanol-to-sample ratio 1 : 4) at 30°C for 2 h. The solution was concentrated at 25°C. Wastes including lipid and chlorophylls were precipitated when the content of ethanol reached approximately 63%. Fucoxanthin was obtained by precipitation when the ethanol concentration reached near 40%. The purity of fucoxanthin was over 90% as examined by high-performance liquid chromatography.

A*β*_1–42_ peptide was purchased from GL Biochem (Shanghai, China). 1,1,1,3,3,3-hexafluoro-2-propanol (HFIP) was obtained from Sigma Chemicals (St. Louis, MO, USA). Soluble A*β* oligomers were prepared as previously described [13, 14]. Briefly, A*β*_1–42_ peptide was dissolved in HFIP to form A*β* monomers. After thoroughly vortexing, 1 mM A*β* monomer solution was aliquoted in 100 *μ*l stock, and stored at −20°C. Milli-Q water (900 *μ*l) was added to 100 *μ*l A*β*_1–42_ solution before the experiments. A*β* solution was further spin-vacuumed and incubated at room temperature for 20 min. HFIP was completely evaporated to obtain the solution of 50 *μ*M A*β*. The A*β* solution was kept at room temperature under constant stirring for 48 h and centrifuged at 14000*g* for 15 min at 4°C. The supernatant (about 900 *μ*l) which contained mainly soluble A*β* oligomers was collected.

SB415286 was obtained from Sigma Chemicals. U0126, wortmannin, and LY294002 were received from LC Laboratories (Woburn, MA, USA). Antibodies were purchased from Cell Signaling Technology (Beverly, MA, USA).

### 2.2. SH-SY5Y Cells Culture

SH-SY5Y cells were maintained in high glucose modified Eagle's medium (DMEM) supplemented with 10% fetal bovine serum and penicillin (100 U/ml)/streptomycin (100 *μ*g/ml) at 37°C with 5% CO_2_. The medium was refreshed every two days. Before experiments, SH-SY5Y cells were seeded in DMEM with 1% fetal bovine serum for 24 h.

### 2.3. Cell Viability Measurements

Cell viability was measured by 3(4,5-dimethylthiazol-2-yl)-2.5-diphenyltetrazolium bromide (MTT) assay based on our previous protocol [15, 16]. Briefly, 10 *μ*l MTT (5 mg/ml) was added to each well in 96-well (100 *μ*l medium/well) plates. Then, plates were incubated at 37°C for 4 h, and 100 *μ*l solvate (0.01 N HCl in 10% SDS) was added. After 16–20 h, the absorbance of samples was measured at a wavelength of 570 nm with 655 nm as the reference wavelength.

### 2.4. Fluorescein Diacetate (FDA)/Propidium Iodide (PI) Double Staining

FDA/PI double staining was performed according to our previous publication [17]. Viable cells were visualized by the fluorescein formed from FDA by esterase activity in the cytoplasm. Nonviable cells were visualized by PI, which only penetrates the membranes of dead cells. Cells were examined after incubation with 10 *μ*g/ml FDA and 5 *μ*g/ml PI at 37°C for 15 min. Images were obtained by UV light microscopy and compared with those taken under a phase-contrast microscopy (Nikon, Tokyo, Japan). To quantitatively evaluate cell viability, images of each well were taken from five randomly selected fields, and the number of FDA-positive and PI-positive cells was counted. The percentage of cell viability was analyzed using the equation % of cell viability = [number of DFA‐cell positive cells / (number of PI‐positive cells + number of DFA‐positive cells)] × 100%.

### 2.5. Hoechst Staining

Chromatin condensation was measured by staining the cell nuclei with Hoechst 33342 as previously described [18, 19]. Cells in 6-well (2 ml medium/well) plates were washed with ice-cold phosphate-buffered saline, fixed, and membrane permeabilized with 4% formaldehyde in 0.1% Triton X-100 for 15 min. Cells were then stained with Hoechst 33342 (5 *μ*g/ml, Thermo Fisher Scientific, Shanghai, China) at 4°C for 5 min. Images were obtained by a fluorescence microscope at 100x magnification (Nikon). To determine the proportion of apoptotic nuclei in each group, images of each well were taken from five randomly selected fields, and the number of pyknotic nuclei and total nuclei was counted. The percentage of pyknotic nuclei was then analyzed using the equation % of pyknotic nuclei = number of pyknotic nuclei / number of total nuclei × 100%.

### 2.6. Intracellular ROS Measurements

The level of intracellular ROS was measured by 5-(and-6)-carboxy-2′,7′-dichiorodihydroflurescein diacetate (carboxy-H_2_DCF-DA, Sigma) as reported in our previous publication [20]. Briefly, cells were washed once with ice-cold phosphate-buffered saline and incubated with 10 *μ*M carboxy-H_2_DCF-DA at 37°C for 10 min. Cells were then washed once with ice-cold phosphate-buffered saline and scanned with a plate reader at 485 nm excitation and 520 nm emission. Images were acquired by a fluorescence microscope (Nikon). Unless otherwise indicated, the fluorescence intensity in SH-SY5Y cells without treatment is expressed as a percentage of the control.

### 2.7. Western Blot Analysis

Western blotting was performed using a well-established protocol [21]. Cell lysates were separated on SDS-polyacrylamide gels and transferred onto polyvinyldifluoride membranes (Pall Corporation, New York, USA). After membrane blocking, proteins were detected by primary antibodies. After incubation at 4°C overnight, signals were obtained after incubation with HRP-conjugated secondary antibodies. Subsequently, blots were developed using the enhanced chemiluminescence plus kit (Beyotime, Hangzhou, China) and signals were exposed.

### 2.8. Statistical Analysis

Results were expressed as mean ± SEM. Differences among groups were compared by analysis of variance (ANOVA) followed by Dunnett's or Tukey's test. *p* < 0.05 was considered as statistically significant.

## 3. Results

### 3.1. Fucoxanthin Effectively Attenuates A*β* Oligomer-Induced Neuronal Apoptosis in SH-SY5Y Cells

**Figure 1:**
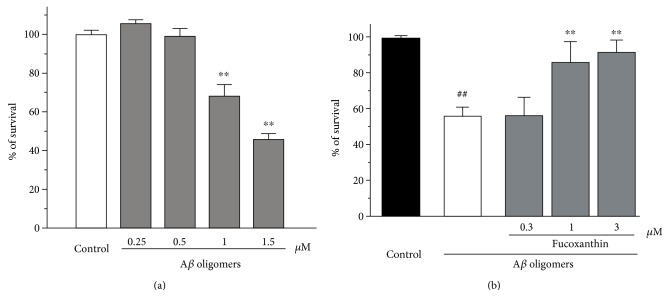
Fucoxanthin attenuated A*β* oligomer-induced neuronal loss as evidenced by the MTT assay. (a) A*β* oligomers induced neuronal loss in a concentration-dependent manner in SH-SY5Y cells. SH-SY5Y cells were treated with A*β* oligomers at various concentrations as indicated. After 24 h, the MTT assay was used to measure cell viability. (b) Fucoxanthin attenuates A*β* oligomer-induced neuronal loss in SH-SY5Y cells. SH-SY5Y cells were treated with fucoxanthin at various concentrations as indicated. After 2 h, 1 *μ*M A*β* oligomer was added. The MTT assay was used to measure cell viability at 24 h after the addition of A*β* oligomers. Data, expressed as percentage of control were the mean ± SEM of three separate experiments; ^##^*p* < 0.01 versus the control group in (b), ^∗∗^*p* < 0.01 versus the control group in (a) and the A*β* oligomer group in (b) (ANOVA and Tukey's test).

We first evaluated the neurotoxicity of A*β* oligomers in SH-SY5Y cells. It was demonstrated that 24 h treatment of A*β* oligomers at concentrations of 1–1.5 *μ*M significantly induced neuronal death in SH-SY5Y cells (Figure 1(a)). Therefore, we used 1 *μ*M A*β* oligomer to induce neurotoxicity in the following study.

To investigate the neuroprotective effects of fucoxanthin, SH-SY5Y cells were pretreated with 0.3–3 *μ*M fucoxanthin for 2 h before adding A*β* oligomers. After 24 h, cell viability was analyzed. Fucoxanthin concentration-dependently attenuated A*β* oligomer-induced reduction of cell viability (Figure 1(b)). Moreover, treatment of 3 *μ*M fucoxanthin alone for 26 h did not induce cell proliferation or cell death (data not shown).

**Figure 2:**
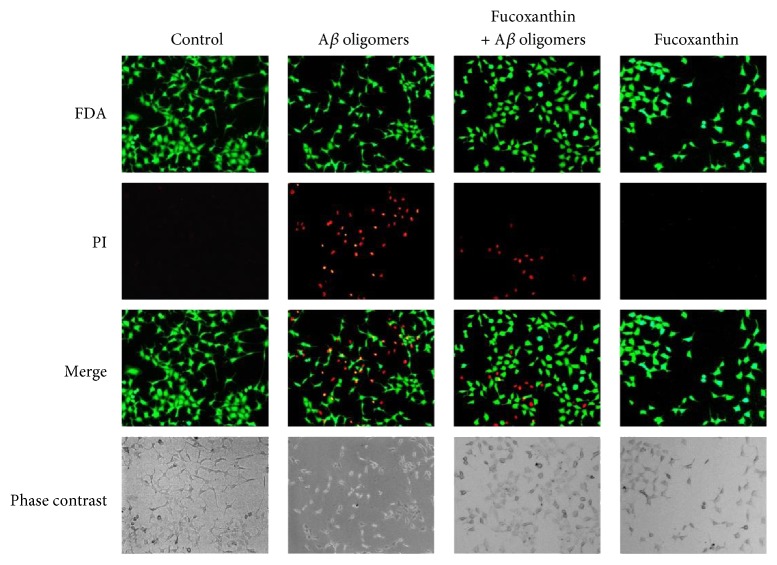
Fucoxanthin attenuates A*β* oligomer-induced neuronal loss as evidenced by FDA/PI double staining. SH-SY5Y cells were treated with 3 *μ*M fucoxanthin. After 2 h, 1 *μ*M A*β* oligomer was added. FDA/PI double staining was used to demonstrate FDA-positive viable cells and PI-positive nonviable cells at 24 h after the treatment of A*β* oligomers.

**Figure 3:**
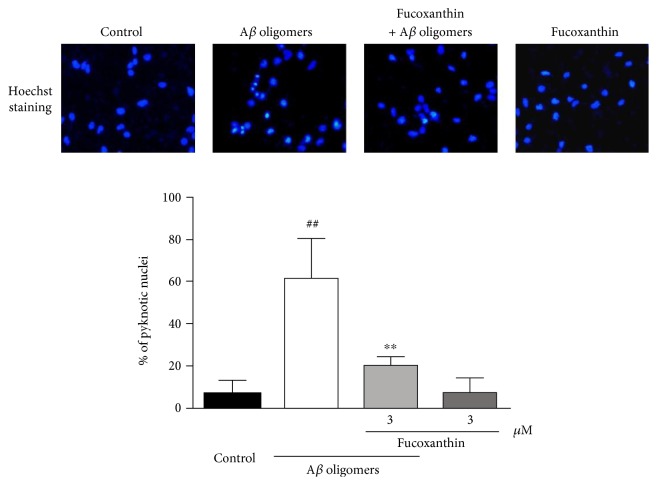
Fucoxanthin attenuates A*β* oligomer-induced neuronal apoptosis as evidenced by Hoechst staining. SH-SY5Y cells were treated with 3 *μ*M fucoxanthin. After 2 h, 1 *μ*M A*β* oligomer was added. Hoechst staining was used to measure the number of pyknotic nuclei with condensed chromatin at 24 h after the treatment of A*β* oligomers. Data were the mean ± SEM of three separate experiments; ^##^*p* < 0.01 versus the control group; ^∗∗^*p* < 0.01 versus the A*β* oligomer group (ANOVA and Tukey's test).

As shown in Figure 2, A*β* oligomers could substantially increase the number of PI-positive nonviable cells and decrease the number of FDA-positive viable cells when compared to the control group. This neurotoxicity of A*β* oligomers was largely prevented by fucoxanthin (Figure 2). Moreover, A*β* oligomers significantly increased the percentage of pyknotic nuclei in SH-SY5Y cells, suggesting that A*β* oligomers caused neuronal loss mainly via inducing cell apoptosis (Figure 3). Furthermore, fucoxanthin significantly decreased A*β* oligomer-induced neuronal apoptosis, as demonstrated by the decrease in the percentage of pyknotic nuclei in the fucoxanthin plus A*β* oligomer group as compared to that in the A*β* oligomer group (Figure 3).

### 3.2. Fucoxanthin Effectively Attenuates A*β* Oligomer-Induced Increase of ROS in SH-SY5Y Cells

**Figure 4:**
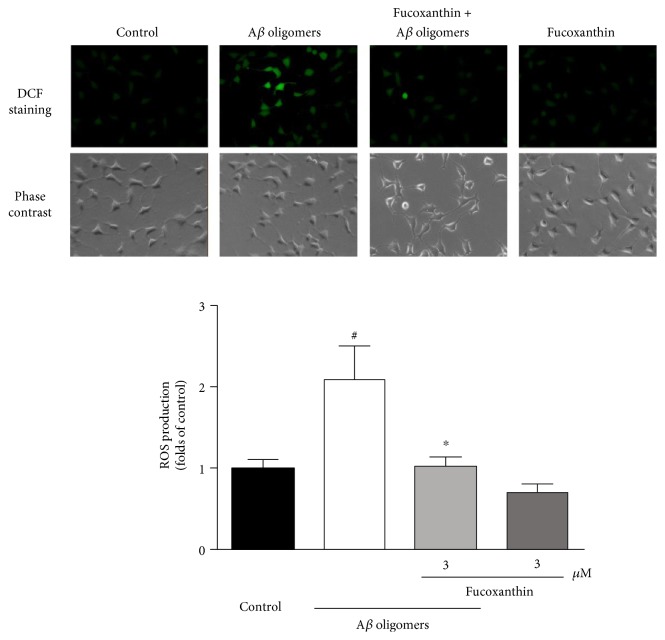
Fucoxanthin attenuates A*β* oligomer-induced increase of intracellular ROS level as evidenced by carboxy-H_2_DCF-DA staining. SH-SY5Y cells were treated with 3 *μ*M fucoxanthin. After 2 h, 1 *μ*M A*β* oligomer was added. Carboxy-H_2_DCF-DA staining was used to measure intracellular ROS level at 24 h after the treatment of A*β* oligomers. Data were the mean ± SEM of three separate experiments; ^#^*p* < 0.05 versus the control group; ^∗^*p* < 0.05 versus the A*β* oligomer group (ANOVA and Tukey's test).

A*β* oligomers significantly increased the level of intracellular ROS in SH-SY5Y cells (Figure 4). This increase in ROS was significantly attenuated by treatment with 3.0 *μ*M fucoxanthin. This finding provides additional support for the neuroprotective effects of fucoxanthin against A*β* oligomer-induced toxicity in SH-SY5Y cells (Figure 4).

### 3.3. Concurrent Activation of the PI3K/Akt Pathway and Inhibition of the ERK Pathway Attenuate A*β* Oligomer-Induced Neuronal Loss in SH-SY5Y Cells

**Figure 5:**
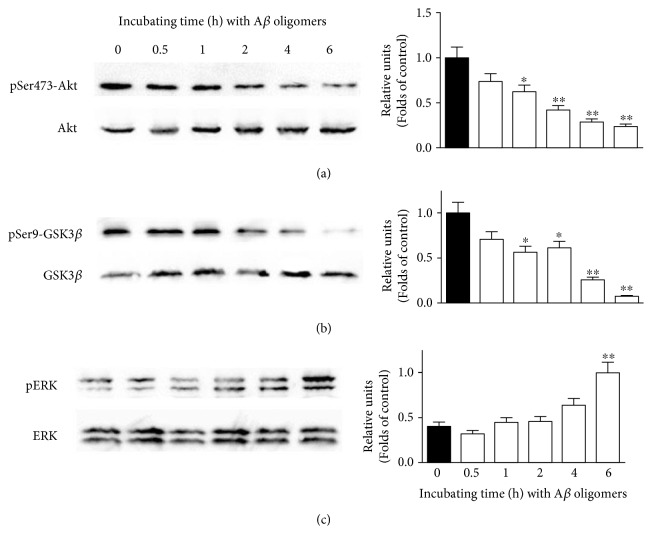
A*β* oligomers inhibit the PI3K/Akt pathway and activate the ERK pathway in SH-SY5Y cells. SH-SY5Y cells were treated with 1 *μ*M A*β* oligomer for various durations as indicated. Western blotting analysis was used to determine the expression of (a) pSer473-Akt, (b) pSer9-GSK3*β*, and (c) pERK. Data were the mean ± SEM of three separate experiments; ^∗^*p* < 0.05 and ^∗∗^*p* < 0.01 versus the control group (ANOVA and Tukey's test).

We further investigated if signaling pathways were involved in A*β* oligomer-induced neuronal loss in our model. We first used Western blotting analysis to explore the alterations of signaling pathways induced by A*β* oligomers. A*β* oligomers were added to SH-SY5Y cells for various durations, and the cellular proteins were extracted. It was demonstrated that A*β* oligomers time-dependently decreased the expressions of pSer473-Akt and pSer9-GSK3*β*, suggesting that A*β* oligomers inhibited the PI3K/Akt pathway (Figures 5(a) and 5(b)). Furthermore, A*β* oligomers also increased the expression of pERK in a time-dependent manner in SH-SY5Y cells, indicating that A*β* oligomers might activate the ERK pathway in our model (Figure 5(c)).

**Figure 6:**
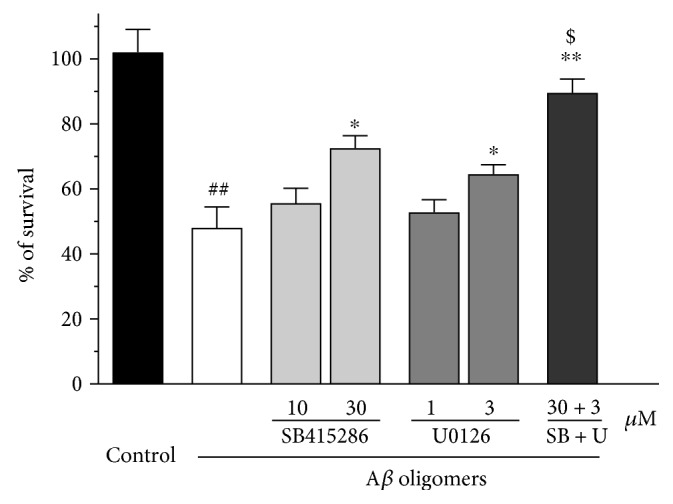
SB415286 and U0126 synergistically attenuates A*β*oligomer-induced neuronal loss in SH-SY5Y cells. SH-SY5Y cells were treated with SB415286, a GSK3*β* inhibitor, and/or U0126, a MEK inhibitor, at various concentrations as indicated. After 2 h, 1 *μ*M A*β* oligomer was added. The MTT assay was used to measure cell viability at 24 h after the treatment of A*β* oligomers. Data, expressed as percentage of control were the mean ± SEM of three separate experiments; ^##^*p* < 0.01 versus the control group, ^∗^*p* < 0.05 and ^∗∗^*p* < 0.01 versus the A*β* oligomer group; ^$^*p* < 0.05 versus the U0126 plus A*β* oligomer group (ANOVA and Tukey's test).

The PI3K/Akt pathway is widely accepted as a prosurvival signaling pathway, while the ERK pathway is generally considered as a proapoptotic pathway [4, 5]. To explore if the regulation of these signaling pathways could attenuate A*β* oligomer-induced neuronal loss, two inhibitors were used. SB415286 is a GSK3*β*-specific inhibitor, while U0126 is a MEK-specific inhibitor. Both SB415286 and U0126 concentrations could dependently attenuated A*β* oligomer-induced neuronal loss in SH-SY5Y cells (Figure 6). Moreover, the combination of SB415286 and U0126 synergistically prevented neurotoxicity induced by A*β* oligomers, suggesting that concurrently activation of the PI3K/Akt pathway and inhibition of the ERK pathway could attenuate A*β* oligomer-induced neuronal loss in SH-SY5Y cells (Figure 6).

### 3.4. Fucoxanthin Prevents the Alterations of the PI3K/Akt and the ERK Pathways Induced by A*β* Oligomers in SH-SY5Y Cells

**Figure 7:**
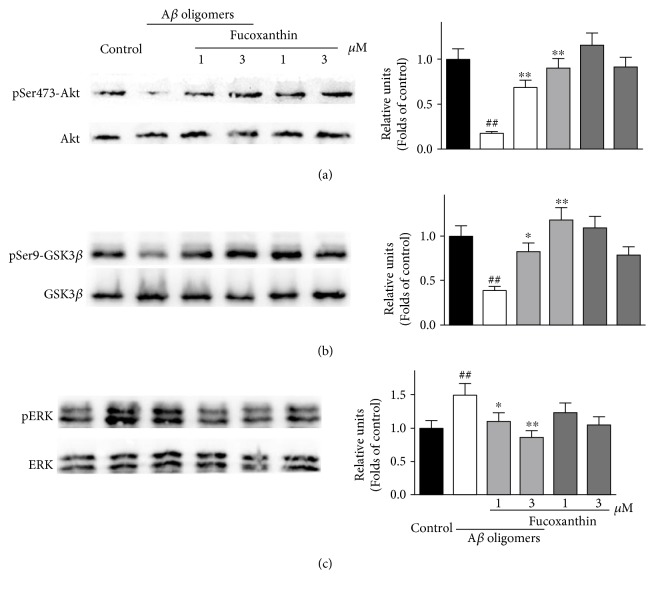
Fucoxanthin prevents A*β* oligomer-induced alteration of the PI3K/Akt and the ERK pathways in a concentration-dependent manner in SH-SY5Y cells. SH-SY5Y cells were treated with 1 or 3 *μ*M fucoxanthin. After 2 h, 1 *μ*M A*β* oligomer was added. Western blotting analysis was used to determine the expression of (a) pSer473-Akt, (b) pSer9-GSK3*β*, and (c) pERK at 6 h after the treatment of A*β* oligomers. Data were the mean ± SEM of three separate experiments; ^##^*p* < 0.01 versus the control group; ^∗^*p* < 0.05 and ^∗∗^*p* < 0.01 versus the A*β* oligomer group (ANOVA and Tukey's test).

To study whether fucoxanthin attenuated A*β* oligomer-induced neuronal loss via regulating the PI3K/Akt and the ERK pathways, we used Western blotting analysis. SH-SY5Y cells were pretreated with 3 *μ*M fucoxanthin for 2 h before adding A*β* oligomers. After 6 h, proteins were extracted. As demonstrated in Figures 7(a) and 7(b), fucoxanthin prevented A*β* oligomer-induced decrease of pSer473-Akt and pSer9-GSK3*β* in a concentration-dependent manner, suggesting that fucoxanthin could attenuate the inhibition of the PI3K/Akt pathway induced by A*β* oligomers. Moreover, fucoxanthin concentration dependently prevented A*β* oligomer-induced increase of pERK, indicating that fucoxanthin also attenuated the activation of the ERK pathway induced by A*β* oligomers (Figure 7(c)). The treatment of fucoxanthin alone did not significantly change the expressions of pSer473-Akt, pSer9-GSK3*β* and pERK (Figure 7).

**Figure 8:**
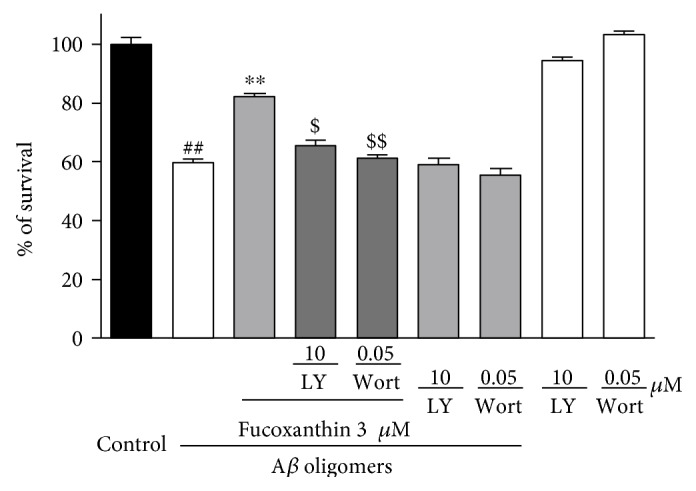
LY294002 and wortmannin significantly abolished the neuroprotective effects of fucoxanthin against A*β* oligomer-induced neuronal loss in SH-SY5Y cells. SH-SY5Y cells were treated with 10 *μ*M LY294002 (LY) or 0.05 *μ*M wortmannin (Wort). After 0.5 h, 3 *μ*M fucoxanthin was added. A*β* oligomers (1 *μ*M) were added at 2 h after the addition of fucoxanthin. The MTT assay was used to measure cell viability at 24 h after the treatment of A*β* oligomers. Data, expressed as percentage of control, were the mean ± SEM of three separate experiments; ^##^*p* < 0.01 versus the control group, ^∗∗^*p* < 0.01 versus the A*β* oligomer group, and ^$^*p* < 0.05 and ^$$^*p* < 0.01 versus the fucoxanthin plus A*β* oligomer group (ANOVA and Tukey's test).

In addition, LY294002 and wortmannin, two PI3K inhibitors were used to confirm if fucoxanthin attenuated A*β* oligomer-induced neuronal loss via regulating the PI3K/Akt pathway. As shown in Figure 8, both PI3K inhibitors significantly abolished the neuroprotective effects of fucoxanthin against neuronal loss induced by A*β* oligomers, providing a support that fucoxanthin produced neuroprotective effects via the activation of PI3K/Akt pathway.

## 4. Discussion

We have reported, for the first time, that fucoxanthin significantly attenuated A*β* oligomer-induced neurotoxicity in SH-SY5Y cells. We further demonstrated that the neuroprotective effects of fucoxanthin against A*β* oligomer-induced neuronal loss and oxidative stress possibly via activating the prosurvival PI3K/Akt pathway and inhibiting the proapoptotic ERK pathway, concurrently.

A*β* oligomers are widely accepted as the main neurotoxins to induce neuronal loss in the brain of AD patients [22]. However, the neurotoxicity of A*β* oligomers formed by different formation protocols varies widely in vitro [23]. In our study, we used a protocol of A*β* oligomer formation which is derived from Roger Anwyl's lab [14, 24]. We found that A*β* oligomers substantially induced neuronal apoptosis at micromolar levels in SH-SY5Y cells, which is consistent with previous publications [25, 26].

We also found that fucoxanthin at 3 *μ*M could significantly attenuate A*β* oligomer-induced neuronal loss in SH-SY5Y cells, as indicated by the MTT assay, FDA/PI double staining and Hoechst staining. These results are consistent with previous publications showing that fucoxanthin at similar concentrations could prevent A*β*-induced neurotoxicity in cortical neurons and microglia [10, 11]. However, in these reports, authors used A*β*_25–35_ or A*β*_1–42_ monomers, which are much less toxic than A*β* oligomers. Moreover, the mechanisms underlying the neurotoxicity of A*β* oligomers and A*β* monomers might be different. Therefore, we further investigated how fucoxanthin attenuated neuronal loss induced by A*β* oligomers. We found that fucoxanthin significantly reversed A*β* oligomer-induced increase of intracellular ROS. Previous studies have shown that there are two hydroxy groups in the ring structure of fucoxanthin that could donate electrons or hydrogen atoms, leading to free radical scavenging and the antioxidative stress properties of fucoxanthin [27, 28]. Therefore, our results provides evidence that fucoxanthin can decrease oxidative stress in neurons, and therefore might be useful in the central nervous systems.

Previous studies have reported that A*β* oligomers could act on both prosurvival and proapoptotic pathways [29, 30]. Therefore, we studied which signaling pathways are mainly involved in A*β* oligomer-induced neuronal loss in our model. We found that (1) both the PI3K/Akt and the ERK pathways were altered by A*β* oligomers and (2) concurrent activation of the PI3K/Akt pathway and inhibition of the ERK pathway synergistically attenuated A*β* oligomer-induced neuronal loss. These results suggested that A*β* oligomers lead to neuronal loss possibly via simultaneous inhibition of the prosurvival PI3K/Akt pathway and activation of the proapoptotic ERK pathway.

We further investigated the regulation of signaling pathways by fucoxanthin. Fucoxanthin attenuated A*β* oligomer-induced decrease of pSer473-Akt and pSer9-GSK3*β*. Moreover, PI3K inhibitors significantly abolished the neuroprotective effects of fucoxanthin. These results suggested that fucoxanthin produced neuroprotective effects at least partially via the activation of the prosurvival PI3K/Akt pathway, which is consistence with a report that fucoxanthin activated the PI3K/Akt cascade to decrease cell injury [31]. Moreover, fucoxanthin attenuated A*β* oligomer-induced increase of pERK in SH-SY5Y cells, indicating that the inhibition of proapoptotic ERK pathway participated in the neuroprotective effects of fucoxanthin. Interestingly, neither SB415286 nor U0126 could fully prevent A*β* oligomer-induced neuronal loss in our model. However, the coapplication of SB415286 and U0126 almost fully reversed neuronal loss induced by A*β* oligomers, which is similar to that of fucoxanthin at 3 *μ*M. These results provided support for the model that fucoxanthin concurrently modulates the PI3K/Akt and ERK pathways to produce neuroprotective effects.

Previous studies reported that oxidative stress could be initiated by many signaling pathways, such as the ERK, the PI3K/Akt, p38, and nuclear factor kappa-light-chain-enhancer of activated B cells nuclear factor (NF-*κ*B) cascade [32]. Besides the ERK and the PI3K pathways, could fucoxanthin act on other molecules to inhibit oxidative stress induced by A*β* oligomers? Akt is reported to act on both GSK3*β* and NF-*κ*B [33]. Therefore, fucoxanthin-induced Akt activation might also regulate NF-*κ*B to inhibit oxidative stress. And whether fucoxanthin could act on other molecules, such as NF-*κ*B, is being investigated in our lab.

Previously, we have reported that fucoxanthin possesses the ability to inhibit acetylcholinesterase [34]. Interestingly, there is a report showing that acetylcholine could decrease the level of extracellular A*β* via activating *α*7 nicotinic acetylcholine receptor (*α*7nAChR) [35]. Moreover, many *α*7nAChR agonists and acetylcholinesterase inhibitors are reported to inhibit A*β* neurotoxicity [36, 37]. Therefore, besides the activation of the signaling pathways, fucoxanthin might also reduce A*β* neurotoxicity via regulating acetylcholine. However, whether fucoxanthin could activate *α*7nAChR is being studied.

Fucoxanthin could be extracted from edible brown seaweeds. Fucoxanthin-rich functional foods are used for antiobesity treatments in Western countries [38]. A double-blind placebo-controlled human study daily using supplementation with 2.4 mg fucoxanthin showed a significantly weight loss in obese women over a 16-week period [39]. No obvious side effects were observed in this study, suggesting that fucoxanthin is quite safe for human use.

In summary, we found that fucoxanthin attenuated A*β* oligomer-induced neurotoxicity and oxidative stress possibly via the activation of the PI3K/Akt pathway and the inhibition of the ERK pathway, concurrently. Based on these findings and the safety of fucoxanthin, we anticipated that further studies of fucoxanthin and related compounds might one day lead to a useful treatment of AD.

## Abbreviations

AD: Alzheimer's disease
ANOVA: Analysis of variance
A*β:*: *β*-amyloid
BACE-1: A*β* precursor protein cleaving enzyme 1
carboxy-H_2_DCF-DA: 5-(and-6)-carboxy-2′,7′-dichiorodihydroflurescein diacetate
ERK: Extracellular signal-regulated kinase
FDA: Fluorescein diacetate
GSK3*β:*: Glycogen synthase kinase 3*β*
HFIP: 1,1,1,3,3,3-Hexafluoro-2-propanol
MEK: Mitogen activated protein kinase
MTT: 3(4,5-Dimethylthiazol-2-yl)-2.5-diphenyltetrazolium bromide
PI: Propidium iodide
PI3K: Phosphoinositide 3-kinase
ROS: Reactive oxygen species
*α*7nAChR: *α*7 Nicotinic acetylcholine receptor.
